# 1,3-Dipolar Cycloaddition of Nitrile Imines and Nitrile Oxides to Exocyclic C=N Bonds—An Approach to *Spiro*-N-Heterocycles

**DOI:** 10.3390/ijms26178673

**Published:** 2025-09-05

**Authors:** Juliana V. Petrova, Maxim E. Kukushkin, Elena K. Beloglazkina

**Affiliations:** 1 Department of Chemistry, M.V. Lomonosov Moscow State University, Leninskie Gory 1-3, 119991 Moscow, Russia; ulenkaiulenka@mail.ru (J.V.P.); lemmingg@mail.ru (M.E.K.); 2 Organic Chemistry Department, RUDN University, Miklukho-Maklaya St. 6, 117198 Moscow, Russia

**Keywords:** 1,3-dipolar cycloaddition, nitrile imines, nitrile oxides, spiro compounds, triazolines, oxadiazolines

## Abstract

Nitrile imines and nitrile oxides are capable of undergoing (3+2)-cycloaddition reactions at double and triple carbon–carbon, carbon-heteroatom, or heteroatom–heteroatom bonds of various dipolarophiles, forming five-membered heterocyclic compounds. When cyclic dipolarophiles bearing an exocyclic carbon–nitrogen double bond (exo-C=N) are introduced into the reaction with these dipoles, spiro-fused 1,2,4-triazoline or 1,2,4-oxadiazoline cycles are formed. Such reactions can provide efficient synthetic approaches to spiro-heterocyclic compounds with enhanced biological activity. This review comprehensively summarizes the literature data on the 1,3-dipolar cycloaddition of nitrile imines and nitrile oxides to exo-C=N bonds for spiro compound synthesis. The research area covers reactions of both saturated and unsaturated dipolarophiles, monocyclic and polycyclic molecules, as well as compounds containing one to three heteroatoms, with special emphasis on systems containing biologically significant heterocyclic pharmacophores. Recent advances in reaction techniques, such as microwave and ultrasonic activation, as well as one-pot and diffusion protocols, are also mentioned.

## 1. Introduction

The most important chemical property of nitrile imines and nitrile oxides is definitely their ability to undergo 1,3-dipolar cycloaddition (or (3+2)-cycloaddition, 32CA) reactions with dipolarophiles, containing carbon–carbon, carbon–heteroatom, or heteroatom–heteroatom multiple bonds forming five-membered heterocycles. Cycloaddition reactions with two last types of dipolarophiles significantly broadens the range of potential synthetic applications of nitrile imines and nitrile oxides compared to some other types of dipoles, which prefer to react with C-C multiple bonds [1]. When a dipolarophile molecule contains several multiple bonds of different nature, the chemoselectivity of the dipolar cycloaddition reaction becomes a significant concern because the activity of 2π-components in relation to nitrile imines and nitrile oxides is known to be comparable for olefins and compounds with a C=N bond [2,3].

Most 1,3-dipolar cycloaddition reactions for nitrile imines and nitrile oxides are effective under non-catalytic conditions and do not require heating or other activation methods, which makes them useful for the reactions with labile substrates. Dipolarophiles with C=C and C=N bonds are convenient for synthetic applications due to their high activity and the superior stability of the resulting heterocycle in comparison, for example, to those formed by the addition of carbon–chalcogen bonds [4].

**Figure 1:**
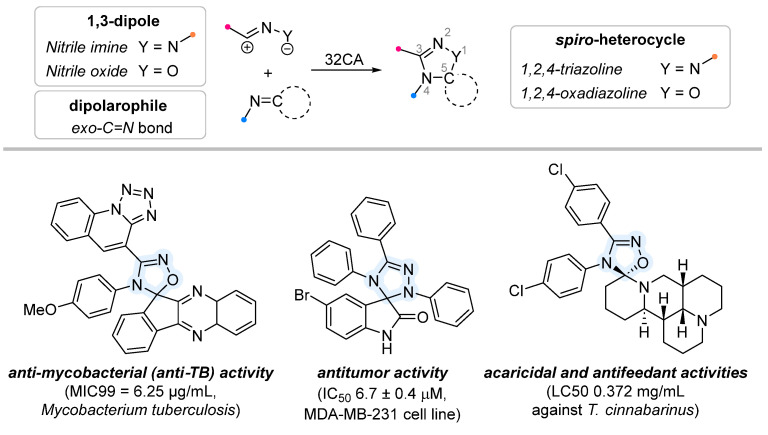
Synthesis of biologically active 1,2,4-triazolines and 1,2,4-oxadiazolines [5,6,7]. The dots of different colors correspond to different substituents R (R = Ar, Alk).

When a 1,1-disubstituted dipolarophile reacts with a 1,3-dipole, a new heterocycle containing quaternary carbon atom is formed. If this atom is part of a cyclic system, and the double bond is exocyclic, then (3+2)-cycloaddition results in the formation of a spiro-conjugated molecule (Figure 1).

**Figure 2:**
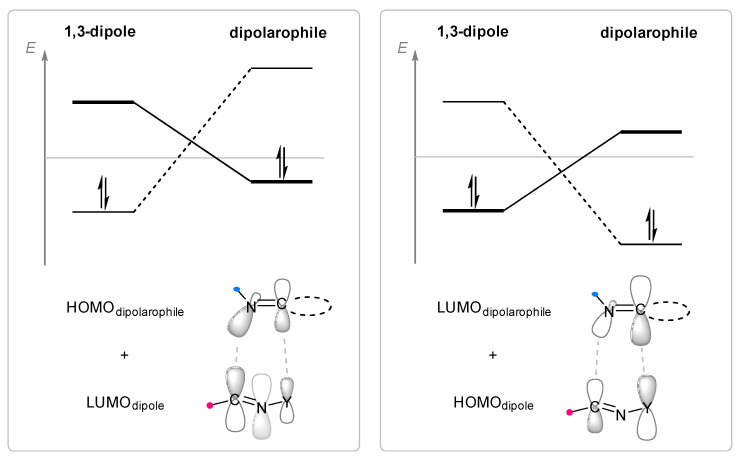
Justification of exceptional regioselectivity: FMO theory rationalization.

Cycloaddition reactions of nitrilimines and nitrile oxides may be described as proceeding through the overlap of the HOMO_dipolarophile_ and the LUMO_dipole_ or vice versa depending on dipolarophile nature [8]. In contrast to olefins, the reactions of the dipolarophilic imino group are significantly more predictable in terms of regiochemistry: nitrile imines and nitrile oxides form five-membered cycles with heteroatoms in the positions 1, 2, and 4 [2,4]. The concept of frontier molecular orbital (FMO) theory allows to rationalize these regioselectivity [9], because the cycloaddition reaction direction is dictated by the distribution of electronic density in the reacting molecules. In other words, it is determined by the ratio of the orbital coefficients on the C and Y atoms of the dipole moiety and those present in the C=N double bond (see Figure 2) [10].

The presence of a quaternary carbon atom in the spiro compounds provide conformational rigidity to their molecules. When some pharmacophoric groups are incorporated into a spiro structure as exocyclic substituents, the geometry of the spirocyclic framework dictates their mutual arrangement. This facilitates the attainment of optimal steric characteristics of molecules for interaction with biological targets. As a result, compounds such as spirooxadiazolines and spirotriazolines are frequently developed as biologically active substances [5,6,7] (Figure 1).

**Figure 3:**
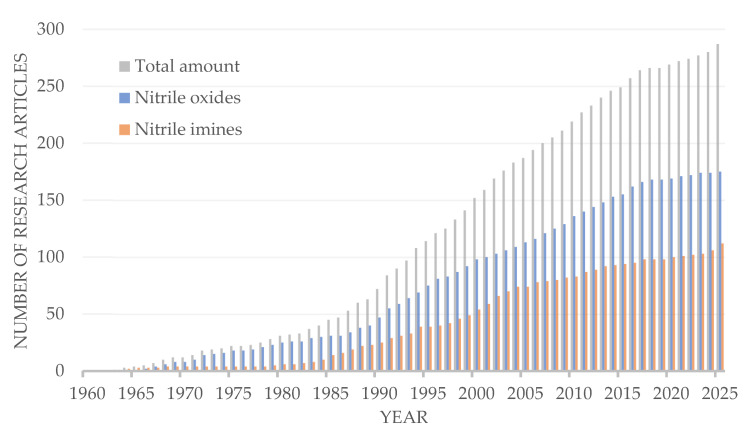
Number of research articles focused on of 1,3-dipolar cycloaddition to C=N double bonds published between 1960 and 2025 (based on the results of a structured search in SciFinder).

These factors have resulted in a continued increase in the number of the articles concerning nitrile imines and nitrile oxides 1,3-dipolar cycloaddition to C=N double bonds (Figure 3). From the time they were first described by Rolf Huisgen [1,11] to the present day, these reactions have become a classical tool in organic chemistry. Note, that there are ~1.5 times as many publications describing the reactions of nitrile oxides as there are for nitrile imines. Of these, approximately one fifth are cycloaddition reactions to exocyclic C=N bonds with the formation of spiro compounds for each dipole type.

The properties of nitrile imines and nitrile oxides were previously discussed in a number of reviews and monographs. Some of them describe the generation methods, main properties, and applications of nitrile imines [2,12] and nitrile oxides [4,13,14]. There are also more specialized papers that present generalized information exclusively about the generation of dipoles [15] or the use of nitrile imines [16] or nitrile oxides [17,18,19] in heterocyclic compounds synthesis. Review articles covering research from a specific period, as shown by references [20,21], are less common. The parts related to (3+2)-cycloaddition reactions can also be found in review articles focused on synthesis of heterocyclic compounds, such as 1,2,4-triazolines [22] and spiro-2-oxindoles [21]. Additionally, it is worth mentioning reviews based on the type of dipolarophile. For example, reactions involving nitrile oxides and carbon–heteroatom bonds [3], the interaction between nitrile imines and multifunctional dipolarophiles [23], and more specifically, reactions of nitrile imines and nitrile oxides with hydrazines, hydrazones, and oximes [24].

Despite the fact that 1,3-dipolar cycloaddition reactions are very effective to the synthesis of spiro compounds, there are currently no works summarizing data on the methods of obtaining and properties of molecules formed by the interaction of nitrile imines or nitrile oxides with dipolarophiles containing exocyclic carbon–nitrogen (exo-C=N) double bonds. The above reviews and monographs provide fragmented information that, when dealing with this topic, undoubtedly poses some difficulties for researchers.

Crossing the equator of the seventh decade of (3+2)-cycloaddition research, we have gathered literature data on methods for synthesis of spiro compounds via cycloaddition of nitrile imines and nitrile oxides to exo-C=N bonds. This review is divided into four main parts according to the type of dipolarophile introduced into the reaction and primarily includes transformations of molecules based on saturated cycles (Section 2.1), which include, in addition to carbocycles, monocyclic and condensed piperidine derivatives. In the second section (Section 2.2) the reactions of nitrile imines and nitrile oxides with exo-C=N bonds of unsaturated dipolarophiles are discussed. This section is the largest in terms of the number of references. Therefore, for convenience, it has been divided into three subsections: monocyclic compounds (Section 2.2.1), condensed carbocycles (Section 2.2.2), and condensed heterocycles (Section 2.2.3). Section 2.2.2 contains only a few examples; Section 2.2.3 is the most extensive compared to the others. The reason for this is the prevalence of cycloaddition reactions described in this part in producing biologically active molecules, whose structure often includes condensed heterocyclic pharmacophore fragments, such as isatin and its derivatives. Reactions of nitrile imines and nitrile oxides with exo-C=N-bonds of monocyclic dipolarophiles are presented in Section 2.3 (cycles with two heteroatoms) and Section 2.4 (cycles with three heteroatoms). The tables, summarizing information regarding the described reactions and their synthetic features, are provided at the end of Section 2.1, Section 2.2.2 (common with Section 2.2.1), Section 2.2.2, Section 2.3 and Section 2.4.

## 2. Cycloaddition to Exocyclic C=N Bonds

### 2.1. Saturated Carbocycles and Piperidine Derivatives with exo-C=N-Bonds in Reactions with Nitrile Imines and Nitrile Oxides

**Figure 4:**
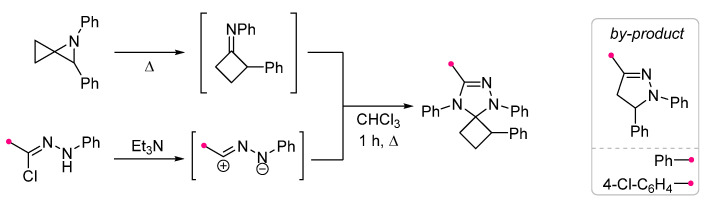
Synthesis of spirocyclobutane using nitrile imine cycloaddition.

The only documented case of the formation of spirocyclobutane as a result of (3+2)-cycloaddition is the reaction of azaspiro[2,2]pentane with diphenyl nitrile imine (DPNI), generated in situ from hydrazonoyl chloride under the action of triethylamine [25]. The formation of this product involves the primary thermally initiated isomerization of azaspiro[2,2]pentane into (phenylimino)cyclobutane, which then interacts with dipole giving a spiro-fused compound. With an increase in the reaction time and the amount of the dipole precursor, the reaction was accompanied by the formation of other final products. Under the conditions shown (Figure 4), only the trisubstituted pyrazoline was formed as a by-product.

**Figure 5:**
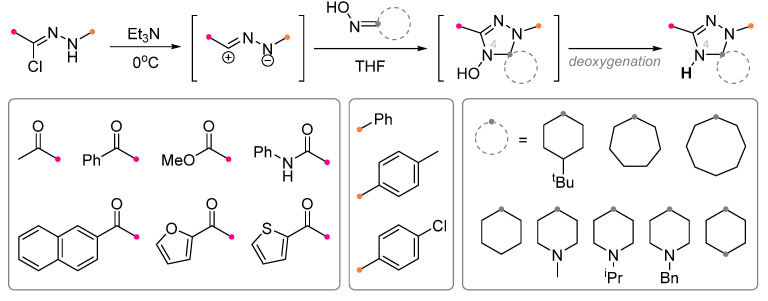
Preparation of N^4^-unsubstituted spirotriazoles from cyclic oximes and nitrile imines.

The properties of oximes and hydrazones with respect to nitrile imines and nitrile oxides were considered in detail in the review [24]; therefore, only the key features of the interaction of these dipolarophiles, important for the synthesis of spiro-conjugated products, will be given here. Despite the commonly occurring similarities in the chemical properties of nitrile imines and nitrile oxides, their interactions with cyclic oximes and hydrazones strongly differ. Under the action of nitrile imines generated from hydrazone chlorides, cyclic oximes were converted into N^4^-unsubstituted spirotriazoles [24,26,27]. It was assumed that the spiro compound with hydroxy group on the N^4^ nitrogen atom was formed as an intermediate. However, due to the presence of excess of triethylamine, the forming compound inevitably lost an oxygen atom (Figure 5). Using this approach, it was also possible to obtain a dispiro-product using dioxime as a starting dipolarophile [28].

It is also known that 4-hydroxy-oxadiazolines, which may be obtained by reacting nitrile oxides with ketoximes, do not undergo deoxygenation and can be isolated [24]. However, there are no known examples of such reactions involving cyclic dipolarophiles, so these reactions will not be discussed in this review.

**Figure 6:**
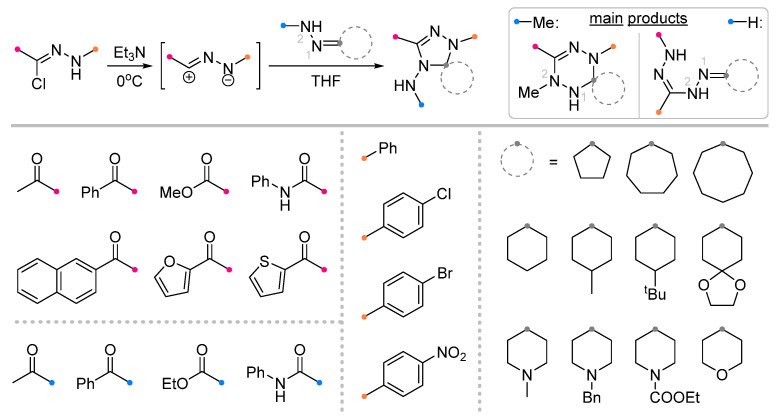
Reactions of cyclic hydrazones and nitrile imines.

The possibility of obtaining spiro-linked triazolines by the reaction of nitrile imines and cyclic hydrazones is limited by the use of dipolarophiles with electron acceptor groups at the terminal nitrogen atom [29] (Figure 6). N^2^-Alkyl cyclohydrazones react with nitrile imines forming spiro tetrahydrotetrazines [24], while N^2^-unsubstituted cycloalkanone hydrazones attach to nitrile imines via nucleophilic addition similar to other amines [2,24].

**Figure 7:**
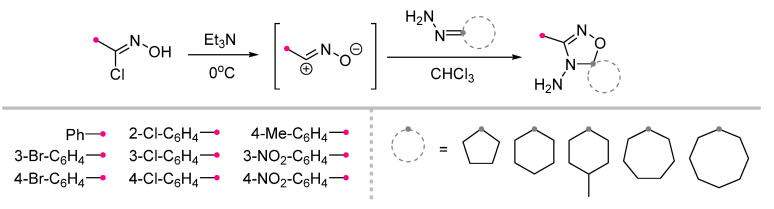
Preparation of spiro-fused 4-aminooxadiazoles from cyclohydrazones and nitrile oxides.

The reactions of nitrile oxides, which are formed in situfrom hydroxyimidoyl halides through the HCl elimination by triethylamine, with cyclic ketohydrazones lead to the formation of spiro-fused 4-aminooxadiazoles [30] (Figure 7). Surprisingly, unlike nitrile imines, the addition of such dipolarophiles occurs nucleophilically only for hydrazones obtained from acetophenone derivatives; in other cases, the product corresponds to the course of a normal (3+2)-cycloaddition [24]. Analyzing the literature data, we found that the number of cyclic hydrazones studied in the reactions with nitrile oxides is significantly lower compared to those tested in reactions with nitrile imines. There is currently no information available on the reactivity of piperidine hydrazones or any reactions of N^2^-substituted cyclohydrazones. The reason may be the low reactivity of the substrates listed, but the authors of the papers on this topic have not specified this.

**Figure 8:**
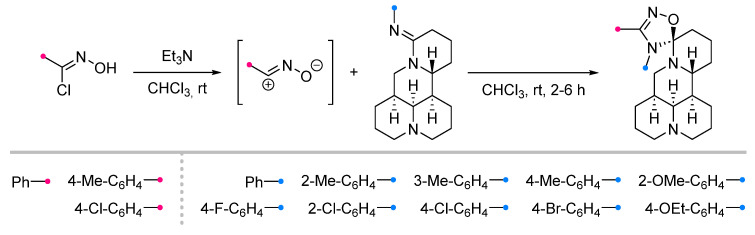
Dipolar cycloaddition of nitrile oxides and imino-substituted matrine-type alkaloids.

One of the rare examples of the nitrile oxides cycloaddition to amidines is the reaction with matrine-type alkaloids [7] (Figure 8). As in the example above, the dipole was formed in situ, and the spiro product was formed not only regio-, but also stereoselectively, due to the pre-determined configuration of the stereocenters in the initial dipolarophile, which was confirmed by the results of X-ray diffraction analysis.

Based on the information provided in this section, it is possible to establish the most common procedure for the cycloaddition reactions of nitrile imines or nitrile oxides with saturated carbocycles and piperidine derivatives containing exo-C=N bonds. The procedure entails the utilization of hydroxyimidoyl or hydrazonoyl halides, which are subsequently converted into the corresponding dipoles by the action of triethylamine. Typically, triethylamine solution was added drop-by-drop under cooling (about 0 °C) to the mixture of dipolarophile and dipole precursor in the same solvent. Then the reaction was conducted at room temperature under stirring. Solvent, reagents ratio, reaction time and isolation technique differ for each substrate, but usually the dipolarophile was taken in an equivalent or greater amount relative to the dipole precursor, when Et_3_N was used in big excess. Tetrahydrofuran (THF) is the most commonly used solvent. Triethylamine hydrochloride, a by-product of dipole generation, is frequently separated through filtration due to its poor solubility in THF. The reaction conditions are summarized in Table 1. Unless noted otherwise, the reactions were carried out under the conditions mentioned above.

### 2.2. Unsaturated Cycles with an Exocyclic C=N Bond as Dipolarophiles in the Reactions with Nitrile Imines and Nitrile Oxides

The presence in the dipolarophile structure of several multiple bonds, accessible for dipole addition, often imposes some limitations on the synthetic applicability of (3+2)-cycloaddition reactions for the preparation of heterocyclic products. Therefore, most of the examples reviewed in this section will be presented by simple cycles containing conjugated double bonds or fused carbo- and heterocycles. It is worth noting that in most of the cited works the reactions of nitrile oxides are described, while for nitrile imines there are only a few examples. In our opinion, this unevenness in the studies may be partly due to the fact that in some cases nitrile oxides are capable of exhibiting greater selectivity in the competition of C=C and C=N bonds [23,31,32].

#### 2.2.1. Reactions of Nitrile Imines and Nitrile Oxides with exo-C=N-Bonds of Monocyclic Compounds

**Figure 9:**
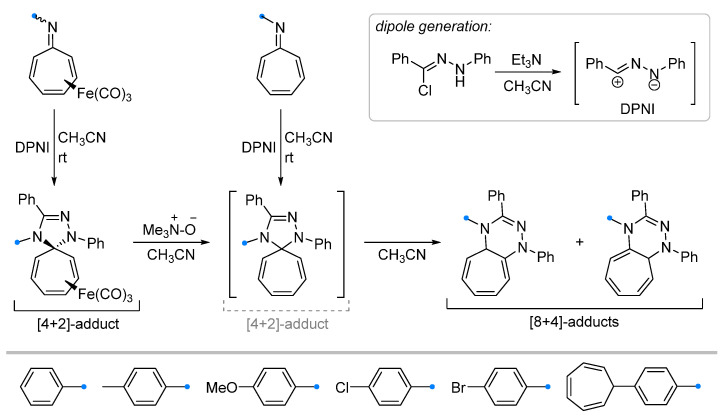
Formation of [4+2]- and [8+4]-adducts of 8-azaheptafulvenes and DPNI.

Investigations of monocyclic unsaturated compounds as dipolarophiles have focused mainly on reactions of 8-azaheptafulvenes (also known as troponimines) and their tricarbonyl iron complexes. It has been shown that spiro-fused cycloheptatrienyl-triazolines can be obtained only for tricarbonyl iron complexes [33]. Reaction of 8-azaheptafulvene itself with DPNI resulted in the formation of the spiro compound only as an intermediate on the way to condensed [8+4]-adducts (Figure 9), which were also formed upon removal of the Fe(CO)_3_-group by the action of trimethylamine oxide on the spiro-fused complex. It is noteworthy that DPNI added to 8-azaheptafulvene complexes only from the side opposite to the Fe(CO)_3_-group, yielding an *anti*-addition product.

**Figure 10:**
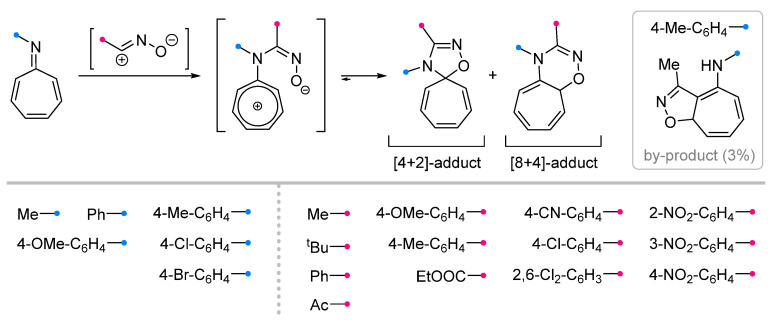
Cycloaddition reactions of nitrile oxides with 8-azaheptafulvenes.

The reactions of nitrile oxides have been studied in a number of works not only on the example of tricarbonyl iron complexes, but also for free 8-azaheptafulvenes. It was shown that, in contrast to the products of nitrile imines addition, spiro-fused cycloheptatrienyl oxadiazolines not only can be isolated, but also for some dipoles (e.g., *tert*-butyl nitrile oxide) are the only products [34,35,36,37]. Nevertheless, the formation of an indivisible mixture of the [4+2]-spiro adduct and the condensed [8+4]-heterocycle is also common [34,35] (Figure 10). The assumption that the reaction occurs with a high degree of asynchrony, and the intermediate zwitterion and the reaction products exist in an equilibrium strongly shifted towards the latter, but sufficient for isomerization, was later confirmed by quantum chemical calculations [38]. A by-product detected in one case indicates that conjugated (3+2)-cycloaddition can occur between nitrile oxide and 8-azaheptafulvene at one of the carbon–carbon bonds, accompanied by isomerization of the exo-imine to the amine [35].

**Figure 11:**
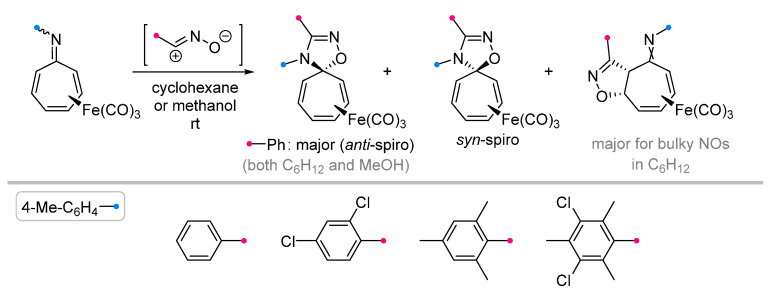
Cycloaddition reactions of nitrile oxides with Fe(CO)_3_-complexes of 8-azaheptafulvenes.

The reactions of Fe(CO)_3_-complexes of 8-azaheptafulvenes with nitrile oxides take place exclusively with the formation of [4+2]-addition products. In addition to *anti-* and *syn*-spiro-complexes, stereoselective formation of a condensed product of (3+2)-cycloaddition to one of the C=C bonds was observed [34] (Figure 11). It was noted that for tricarbonyl iron complexes, the isomerization of the main *anti*-spiro product into *syn*-spiro product is also possible in hexafluoroisopropanol and other highly polar solvents that stabilize the intermediate zwitterion [39]. Unfortunately, an assessment of the applicability of this method for the synthesis of spiro compounds is not possible, since no information on the isolation of individual spiro compounds and other reaction products is described in [34,39].

**Figure 12:**
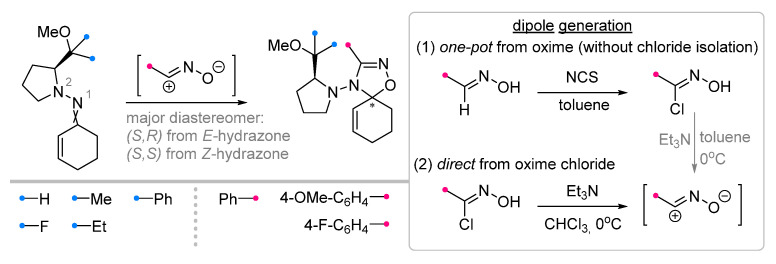
Diastereoselective synthesis of spirooxadiazolines from cyclohexenone hydrazones.

Cyclohexenone hydrazones containing chiral substituents at the N^2^ atom were reacted with nitrile oxides under different conditions [40] (Figure 12); the main difference being the dipole precursor used. It was shown that the use of a procedure that does not involve the isolation of chlorooxime is less efficient than direct generation of nitrile oxide from the corresponding hydrochloride. The diastereoselectivity of the reaction was assessed independently for E- and Z-hydrazones; the latter allowed to prepare spirooxadiazolines with higher diastereomeric excess values.

It was shown in the work [41] that stable nitrile oxides are capable of adding to the imino form of aminopyridines to form a spiro compound corresponding to (3+2)-cycloaddition to the exocyclic C=NH bond. Unfortunately, the resulting product cannot be isolated, so the presented reaction is not of preparative interest for obtaining spiro compounds.

#### 2.2.2. Addition of Nitrile Oxides and Nitrile Imines at the Exocyclic C=N Bond of Fused Carbocycles

**Figure 13:**
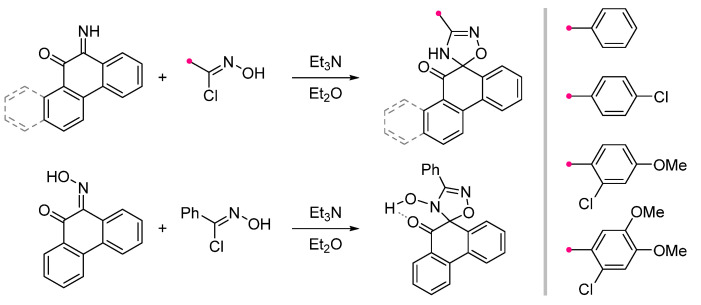
Reactions of nitrile oxides and imines of phenanthraquinone and chrysenequinone.

Cycloaddition reactions at exocyclic C=N bonds of unsaturated condensed carbocyclic compounds are poorly presented in the literature. Only a few examples of nitrile oxide reactions involving imines of phenanthraquinone and chrysenquinone, as well as phenanthraquinone oxime, are known [42,43] (Figure 13). As for the reaction of nitrile oxides with ketoximes [24], the spiro-fused derivative of phenanthraquinone retains the N^4^-hydroxygroup. The formation of a hydrogen bond between it and the carbonyl group located near the spiro fusion have been observed (detected by IR spectroscopy) [43].

To summarize, it should be noted that the reactions between nitrile imines or nitrile oxides and unsaturated carbocyclic compounds containing exo-C=N bonds do not always lead to the formation of spiro conjugated products (Table 2). The formation of 1,2,4-oxadiazoline products was more prevalent than 1,2,4-triazoline products. Probably, the steric characteristics of the dipole had a great influence on the cycloaddition course. Thus, in the reaction with DPNI, azaheptafulvenes yielded only [8+4]-products, and under the action of nitrile oxide, it was possible to obtain a spiro-conjugated [4+2]-product. When interacting with bulky nitrile oxides, such as mesitonitrile oxide, even azaheptafulvene complexes lost their ability to act as C=N dipolarophiles and reacted via C=C reaction center.

Unlike the reaction conditions for saturated carbocycles (THF was used as a solvent and a dipole was generated upon cooling), the reactions of unsaturated dipolarophiles are more difficult to standardize. Both methods that involve the gradual addition of one component (a base solution or a dipole precursor) and those that involve mixing all components simultaneously are employed. It is also worth noting that the dipole precursor is often used in excess relative to the dipolarophile in these reactions. Studies of the reactions of sufficiently stable benzonitrile oxides have shown that it is possible to use a dipole on its own, rather than generating it in situ. In most cases, the target spiro compounds were obtained in good yield (Table 2).

#### 2.2.3. Reactions of Nitrile Oxides and Nitrile Imines at Exocyclic C=N Bonds of Fused Heterocycles

**Figure 14:**
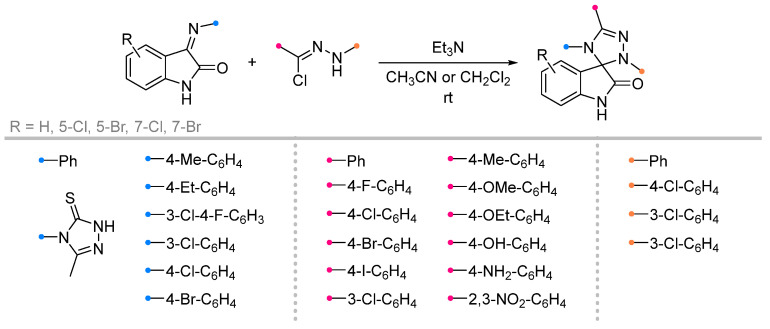
Synthesis of spiroindolinones from isatin derivatives and nitrile imines.

A wide range of biologically active spiroindolinones have been synthesized by the action of nitrile imines generated in situ from hydrazonoyl halides and imines previously obtained from isatin [6,44]. The reactions proceeded in the usual manner, allowing the preparation of spiro-fused triazolines in good yield (Figure 14). Several equally effective techniques are known, which involve microwave action to accelerate the reaction [45,46]. The methods used showed high tolerance to various substituents among the aromatic groups of the nitrile imine and dipolarophile.

**Figure 15:**
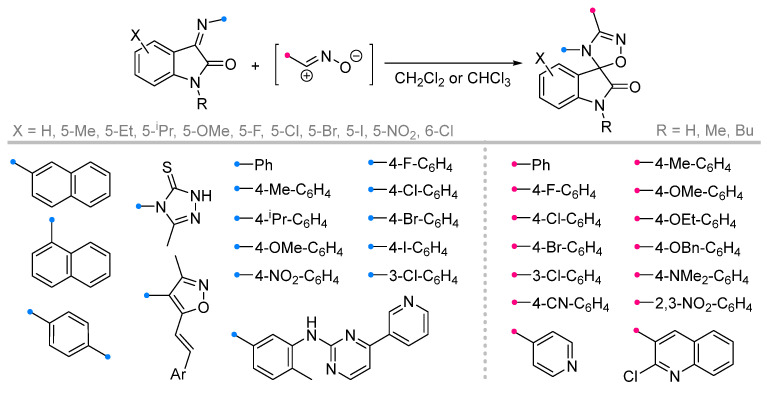
Synthesis of spiroindolinones from isatin derivatives and nitrile oxides.

Quite widely represented are the studies of iminoindolinones in reactions with nitrile oxides (Figure 15). The diversity of substrates in this case is supported by the diversity of the used methodologies. Along with traditional approaches [47,48,49,50], some methods with one-pot dipole generation from benzaldoxime (similar to that shown in Figure 12) [51,52] or one-pot synthesis of the spiro compounds from isatin and amine [53], ultrasonic activation [54] or in a catalytic version are also used [53]. The existence of many methods complicates the definition of uniform conditions for this type of reaction; however, the most common for these transformations should be considered chlorinated solvents (CH_2_Cl_2_ and CHCl_3_), as well as their mixtures with other solvents designed to increase the solubility of the reagents. It was shown that the reactions proceed efficiently for the dipoles with the substituents in the aromatic rings with different electronic effects, as well as for sterically hindered imines and diimines.

**Figure 16:**
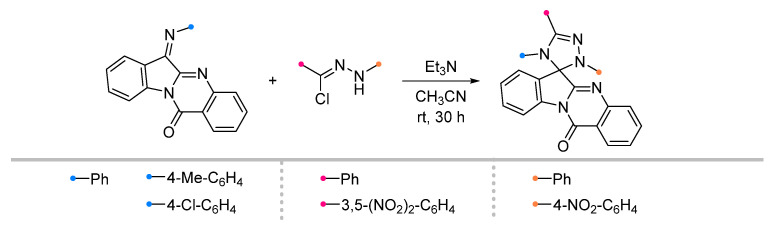
Triazoline-tryptanthrin spiro compounds formation.

Arylimines, which are derivatives of tryptanthrin (or indoloquinazoline), are capable of adding nitrile imines similarly to other indoles and their derivatives [55] (Figure 16). The preparation of spirotriazolines in this manner does not require special conditions, and the target products are formed after 30 h in good and excellent yields.

**Figure 17:**
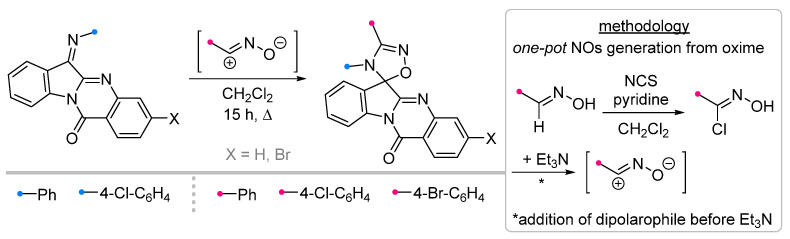
Oxadiazoline-tryptanthrin spiro compounds formation.

One of the most successful examples of the use of the one-pot strategy in the synthesis of spiro compounds is the transformation of tryptanthrin imines under the nitrile oxide action. Unlike many approaches used in (3+2)-cecloaddition reactions, the reaction described in [56] occurs at elevated temperature, resulting in the formation of the target product in high yield (Figure 17).

**Figure 18:**
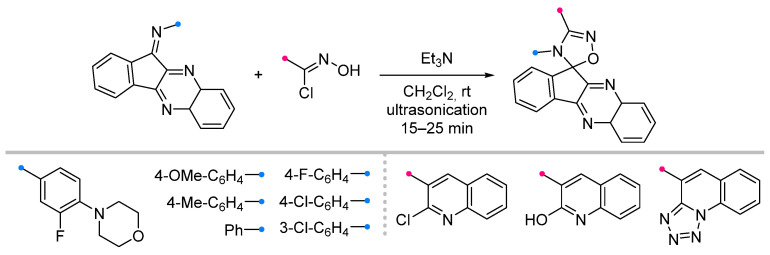
Ultrasonic-activated reactions of nitrile oxides and with fused imines.

In a recent study [5] it was shown that with ultrasonic activation of the reactions of nitrile oxides with fused imine can be reduced the reaction time to 15–25min without reducing the yield of the spiro products (Figure 18).

Thus, the methods of producing spiro compounds from condensed heterocyclic dipolarophiles are highly diverse. In all cases, the interaction of nitrile imines or nitrile oxides and exo-C=N bonds resulted in the formation of spiro-conjugated products with good to excellent yields (Table 3). Special attention should be given to one-pot methodologies, which can enhance the efficiency of synthesis by reducing the number of stages, as well as reactions that are induced by microwaves or ultrasound, which require only a few minutes to complete.

To summarize, these studies show that nitrile imines or nitrile oxides react with fused heterocyclescontainingexo-C=N-bonds. Functional groups in these heterocycles are much less reactive than simple C=N bonds. Therefore, the regioselectivity of the cycloaddition reactions was discussed even more often in these papers than, for example, chemoselectivity, which received a lot of attention in the articles from the previous section.

### 2.3. Addition Reactions of Nitrile Imines and Nitrile Oxides to the Exocyclic C=N Bonds of Cycles with Two Heteroatoms

**Figure 19:**
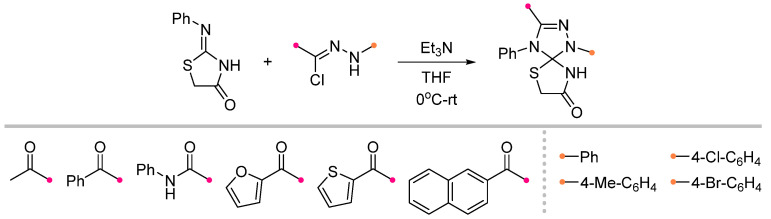
Synthesis of spiro compounds from 2-imino-thiazolidin-4-ones and nitrile imines.

The reaction of 2-imino-thiazolidin-4-one and nitrile imine proceeds according to the classical scheme with the formation of a spiro-fused product [57]. As in the case of the above (3+2)-cycloaddition reactions, the dipole chemoselectively adds to the C=N bond without affecting the exocyclic C=O group (Figure 19).

**Figure 20:**
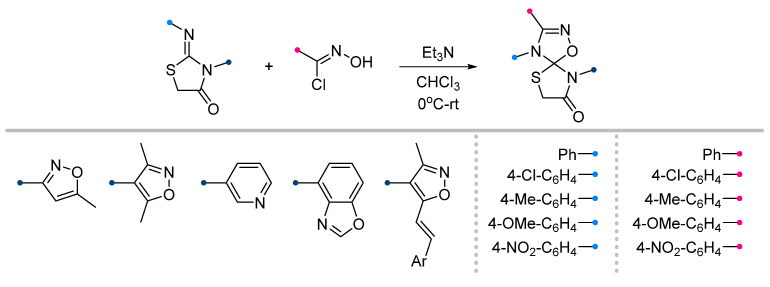
Synthesis of spiro compounds from 2-imino-thiazolidin-4-ones and nitrile oxides.

The amide C=O bond remains inert when these dipolarophiles are reacted with nitrile oxides (Figure 20). It is surprising that, in contrast to the nitrile imine reactions, which have been studied for thiazolidin-4-ones unsubstituted at the N^3^ nitrogen atom, the addition of nitrile oxide has only been described for dipolarophiles containing heteroaromatic substituents at this position, such as oxazoles [58,59,60], benzoxazole [61] and pyridine [62]. The reactions are carried out in a traditional manner, which demonstrates high efficiency for all of the listed substituents.

**Figure 21:**
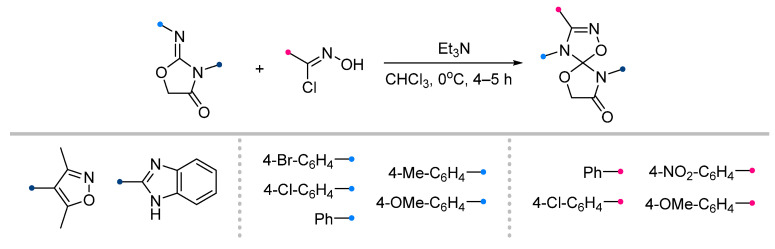
Formation of oxazolidine-oxadiazoline spiro compounds from nitrile oxides.

Despite the structural similarity, only two examples of the use of 2-imino-oxazolidin-4-ones in the reactions of (3+2)-cycloaddition are known (Figure 21). As in the previous case, only reactions of aromatic nitrile oxides and dipolarophiles with heterocyclic substituents at the N^3^ atom of oxazolidine were investigated [60,63]. Imino-oxazolidin-4-ones were introduced into (3+2)-cycloaddition reactions under conditions similar to thiazolidines (under cooling in CHCl_3_) giving spiro-fused oxadiazolines with yields more than 70%.

**Figure 22:**
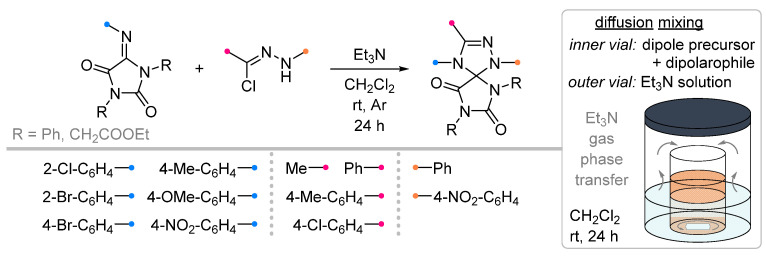
1,3-Dipolar cycloaddition of nitrile imines to iminohydantoins.

To obtain spiro-fused hydantoins (imidazolidine-2,4-diones) with the triazoline ring, the corresponding arylimines were introduced into the reaction with nitrile imines (Figure 22) [64]. The dipole was generated in the classical way from hydrazonoyl halide and triethylamine. The latter was introduced into the reaction mixture by two methods: by dropwise addition (the most common method) and via diffusion through the gas phase. Both methods were shown to be very effective; the yields of the products depended to a greater extent on the electronic characteristics of the substituents in the aromatic fragments and, more unexpectedly, on the nature of the substituents at the N^1^ and N^3^ atoms of the imidazolidine (R in Figure 22).

**Figure 23:**
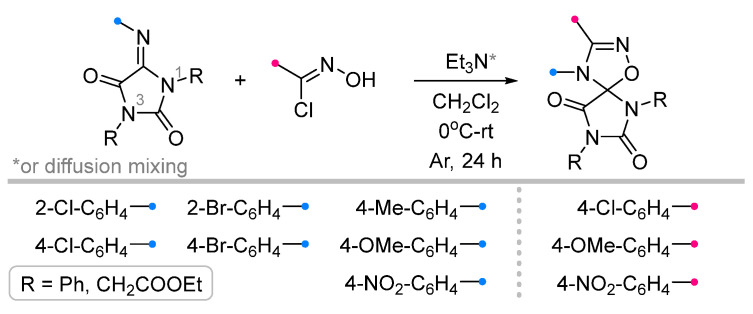
Dipolar cycloaddition of nitrile oxides and iminohydantoins.

Both under conventional conditions and the diffusion approach, iminohydantoins were reacted with nitrile oxides (Figure 23) [65]. In contrast to the above nitrile imine reactions, diffusion mixing of the reactants was in some cases significantly inferior to classical conditions, in which the target spiro compounds were formed in good or excellent yields. At the same time, it was found that the presented method has a number of limitations: in addition to the low reactivity of N^1^-alkyl iminohydantoins (similar to that presented for nitrile imines), the (3+2)-cycloaddition reaction did not occur for carbethoxyformonitrile oxide (EtOOC-CNO) even for N^1^,N^3^-bis-aryl substituted dipolarophile.

**Figure 24:**
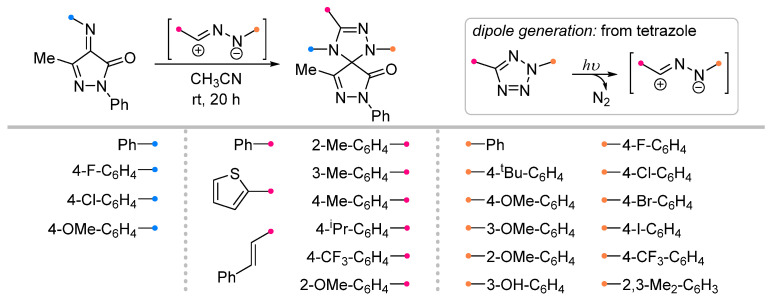
Synthesis of spiro-fused triazolines from nitrile imines generated from tetrazoles.

The work [66], describing the synthesis of spiro-fused triazolines by the reaction of nitrile imines and 4-imino-pyrazolin-3-ones, is the only example of (3+2)-cycloaddition of these dipoles with an exo-C=N bond initiated by the action of light (Figure 24). The authors of the article chose tetrazoles as dipole precursors, which were photochemically converted into nitrile imines with the release of molecular nitrogen. It was shown on a large number of substrates that the selected conditions allow the synthesis of spiro compounds with excellent yields.

To sum up, in most of the examples presented in this section, the dipolarophile and the dipole precursor were used in equal or similar quantities (Table 4). The “traditional” methods assumed the formation of nitrile oxide under cooling, nitrile imines were generated at room temperature. Alternative techniques, such as diffusion mixing of reagents or dipole photogeneration, were applicable for both the synthesis of 1,2,4-triazolines and 1,2,4-oxadiazolines. Despite this, for the substrates discussed in this section, there are currently no examples of the catalysts use or other activation methods, such as microwave or ultrasound. The reaction time ranges from several hours to a day.

It is noteworthy that all the articles in this section were published within the last 15 years, which demonstrates the relevance of the subject matter.

### 2.4. Nitrile Imine and Nitrile Oxide Reactions at Exocyclic C=N Bonds of Heterocycles with Three Heteroatoms

**Figure 25:**
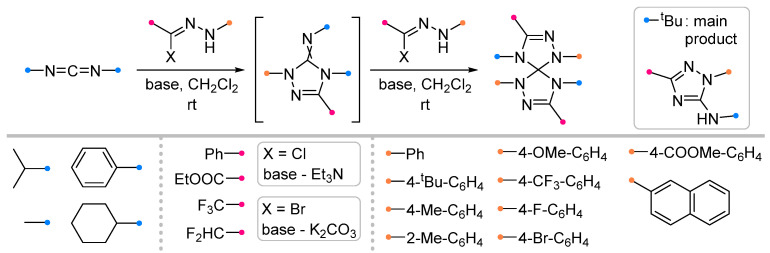
Carbodiimide synthesis of bis-1,2,4-triazolinespiranes.

The applicability of the method first described in 1965 [67] for the synthesis of bis-1,2,4-triazolinespiranes at the reactions of nitrile imines with carbodiimides has recently been extended to various fluorine-containing dipoles [68] (Figure 25). It was shown that the preparation of similar spiro products by the addition of two different dipoles is also possible, but it requires the synthesis of the intermediate imino-triazoline by an alternative method [67]. Being a more active dipolarophile than carbodiimide, this intermediate adds a second dipole molecule more quickly and therefore cannot be isolated. It was also noted that the addition of nitrile imine to an imino-triazoline with an exo-C=NH group does not allow to synthesise the spiro-fused product, which apparently turns out to be unstable [69]. The method also had other limitations, for example, di-*p*-tolyl-carbodiimide did not react with the CHF_2_-substituted dipole, and when using di-*tert*-butyl-carbodiimide, the main product was aminotriazole [68], which was also formed from other carbodiimides when boiling them with nitrile imine in toluene [70].

**Figure 26:**
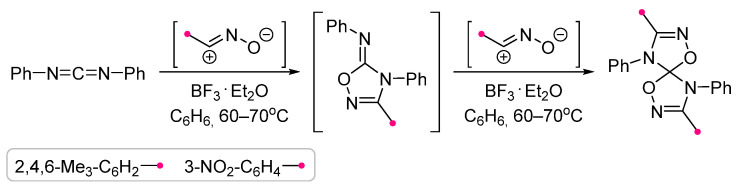
Carbodiimide synthesis of bis-1,2,4-oxadiazolinespiranes.

The reactions of nitrile oxides with carbodiimides were similar to the example presented above, but in the presence of boron trifluoride etherate, which was necessary for the activation of the C=N bond in the 1,3-dipolar cycloadition reaction (Figure 26) [71]. It is worth noting that under non-catalytic conditions nitrile oxide did not add to diphenylcarbodiimide even after many hours of heating in benzene. The addition of boron trifluoride made it possible to obtain the spiro-fused product in less than an hour. As for nitrile imines, obtaining a structure formally corresponding to the addition of two different dipole molecules was possible only if the intermediate imino-oxazolidine was synthesized by an independent method.

**Figure 27:**
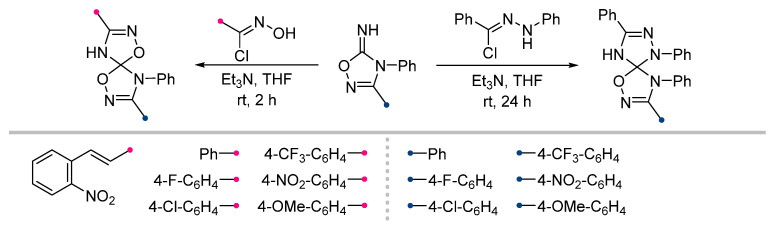
Oxadiazoline imine—precursor for triazoline-oxadiazoline and bis-oxadiazoline spir compounds.

A more convenient dipolarophile for the synthesis of spiro compounds of the oxadiazoline-oxadiazoline type and the only precursor of triazoline-oxadiazolines was oxadiazole imine, the reactions of which with nitrile imines and nitrile oxides were described in [72] (Figure 27). The introduction of exo-C=N-unsubstituted oxadiazoline imine into the (3+2)-cycloaddition reactions made it possible not only to carry out reactions with nitrile oxides in the absence of a catalyst, but also to significantly reduce the reaction time compared to that required for nitrile imines. The reactions of nitrile oxides were also performed through the fluoride ion activation and in absence of any additives. When CsF/18-crown-6 was used as the fluoride ion source for dehydrochlorination of hydroxyimidoyl halide and dipole generation, cycloaddition reactions proceeded almost as efficiently as when using the base. Experiments conducted in a self-catalyzed manner (without base or F^−^-source additives) provided spiro compounds in low to moderate yields (29–56%) because dipolarophiles in this case were also forced to act as a base. In contrast to unstable triazoline-triazolines, both triazoline-oxadiazoline and bis-oxadiazoline spiro compounds synthesized from this dipolarophile were stable enough for isolation, characterization, and study of their biological properties.

In the articles mentioned in this section, reactions were carried out under various conditions. Since it was described in detail in the text above, Table 5 only contains information about general procedures.

The works presented in this section may be examples of the successful revitalization of studies which were performed during the early stages of the development of 1,3-dipolar cycloaddition reactions. Being previously known only in rare cases, these reactions have now become widely used and effectively applied for the synthesis of biologically active compounds. Insights into the reactivity patterns of the substrates studied in this context undoubtedly contribute significantly to the advancement of chemistry of heterocyclic compounds.

## 3. Conclusions

The synthesis of compounds with a spiro-fused framework via the (3+2)-cycloaddition reaction at exocyclic carbon–nitrogen bonds appears to be not only feasible but also a convenient synthetic approach to these derivatives. These reactions are highly predictable in terms of regio- and chemoreactivity, and in most cases, they do not require the use of catalysts or specific conditions for efficient synthesis. Probably, the existence of these features is the reason for the low implementation of currently activelydeveloped approaches, for example, flow reactions. Due to the lack of other possible pathways, these transformations become the object of computer modeling incomparably rarely relative to other 1,3-dipolar cycloaddition reactions. In addition to the traditional synthesis method, there are only a few alternative approaches that are used: microwave and ultrasonic activation, one-pot and diffusion techniques. Because the primary goal of research is seldom to produce optically active compounds, there are no known examples of the use of chiral catalysts in these reactions.

It is worth noting that, at the moment, conditions in which endocyclic carbon–nitrogen bonds often exhibit good activity have not been employed towards developeingexo-C=N dipolarophiles. Such conditions include, for instance, those that contribute to the formation of bis-adducts in reactions with other dipolarophiles. This refers to reactions in which a dipole is first attached to a dipolarophile, and then to the resulting heterocycle. Such examples are not unusual for both nitrile imines and nitrile oxides. These reactions occur even in the absence of a base in an aqueous buffer [73]. However, this has not been adapted for exo-C=N dipolarophiles.

Despite the limited development of alternative techniques, the effectiveness and usability of established synthesis methods should not be underestimated. The versatility of classical methods of (3+2) cycloaddition has been demonstrated by numerous studies of compounds with an exo-C=N bond as dipolarophiles, such as cyclic imines, oximes, hydrazones, amidines, etc. Spiro compounds formed by dipolarophiles of a polycondensed structure or complex compounds reflect the high reactivity of nitrile imines and nitrile oxides even toward sterically hindered substrates.

## Figures and Tables

**Table 1:** Summary of substrates, conditions and yields of the reactions of nitrile imines or nitrile oxides and saturated carbocycles and piperidine with exo-C=N-bonds.

Substrate	Dipole	Reagents Ratio ^a^	Time	Yields, %	Note	Refs.
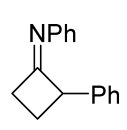	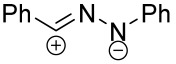	1/1/5	1 h	54	Reactions were conducted in CHCl_3_ under reflux, dipole precursor was added dropwise to the mixture of dipolarophile precursor and Et_3_N	[25]
	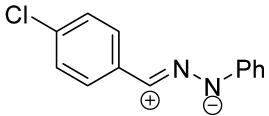	1/2/10	4 h	38		
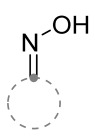	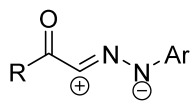	3/1/6	12 h	52–72	R = methyl, methoxy, aryl, anilino ^b^	[27]
		2.5/1/5	12 h	48–62	R = anilino, heteroaryl; 1,4-Dioxane was used as the solvent ^b^	[26]
		3/1.5/5	overnight	40–58	R = methyl, aryl	[24]
		1/2/2	overnight	45–54	R = methyl, methoxy, aryl, anilino; Dipolarophile was 1,4-cyclohexanedione dioxime	[28]
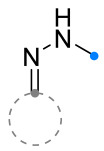	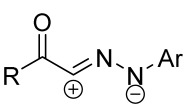	3/1.5/5	overnight	30–87	R = methyl	[24]
		2/1.5/5	overnight	25–87	R = methyl, methoxy	
		1/1/5	4–6 h	50–86	R = aryl	
		2/1/5	overnight	70–90	R = anilino, aryl, heteroaryl ^b^	[29]
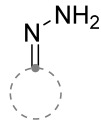	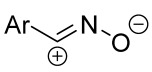	2.5/1/2.5	2 h	38–80	Reactions were conducted in CHCl_3_, dipole precursor was added dropwise to the mixture of dipolarophile and Et_3_N.	[24] [30]
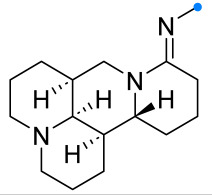	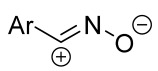	1/1/2	2–6 h	25–82	Reactions in CHCl_3_^b^	[7]

^a^ The ratio of dipolarophile/dipole precursor/triethylamine, in equivalents; ^b^ Reactions were carried out at room temperature.

**Table 2:** Summary of substrates, conditions and yields of the reactions of nitrile imines or nitrile oxides and unsaturated carbocycles with exo-C=N-bonds.

Substrate	Dipole	Reagents Ratio ^a^	Conditions	Total Yields and Selectivity ^b^, %	Note	Refs.
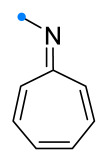	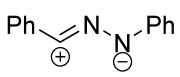	1/1/3	CH_3_CN, rt, 5 h	>90 (100)	Only [8+4]-adducts were obtained. None of the reagents were added gradually.	[33]
	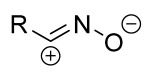	1/1.1/1.25	C_6_H_6_, rt, ~12 h ^c^	50–90 (0–100)	R = alkyl, aryl. Substituents in both reagents had an impact on the preference between [4+2]- and [8+4]-products.	[35]
		1/1.2	CH_3_OH, rt, 1–15 h, Ar	85–90 (100)	Only spiro products were obtained. Benzonitrile oxides were used themselves.	[34]
		1/2	rt, 30 min ^d^	77–98 (100)		[36] [37]
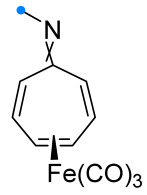	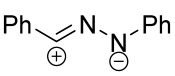	1/1/3	CH_3_CN, rt, 6 h	>80 (100)	Only spiro products were obtained. None of the reagents were added gradually.	[33]
	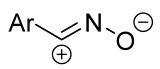	no data	CH_3_OH, rt ^e^	≥95 (65–100)	Major [4+2]-product is spiro compound (mixture of *syn*- and *anti*-), minor is fused (C=C).	[34]
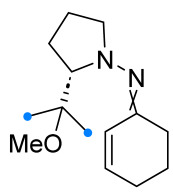	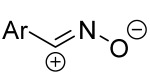	1/2/2	CHCl_3_, 0 °C to rt, 6–8 h	49–87 (100)	Only spiro products were obtained as two diastereomers: (S,R) from E-hydrazone (*de* = 28–86%), (S,S) from Z-hydrazone (*de* = 5–98%). The dipole precursor was gradually added to the dipolarophile and Et_3_N.	[40]
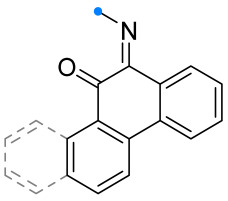	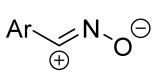	see Note column	Et_2_O, reflux, 2 h	55–82 (100)	Only masses of reagents were given: 1 g of imine and 10 g of hydroxybenzimidoyl chloride [42], 1 g of imine and 7 g of hydroxyimidoyl chloride [43], 1 g of oxime and 5 g of hydroxyimidoyl chloride [43].	[42] [43]

^a^ The ratio of dipolarophile/dipole precursor/triethylamine, in equivalents; ^b^ The parameter demonstrates an excess of one product relative to another if the reaction is accompanied by the formation of several cycloaddition products (other by-products are not taken into account). Calculated as the subtraction of the yields of the major and minor products divided by the total yield (×100%); ^c^ A solution of the dipole precursor (1.1 equiv.) and a solution of Et_3_N (1.1 equiv.) were added at the same time to the mixture of dipolarophile (1 equiv.) and Et_3_N (0.15 equiv.); ^d^ The solvent was not mentioned; ^e^ Reaction time was not specified.

**Table 3:** Summary of substrates, conditions and yields of the reactions of nitrile imines or nitrile oxides and fused heterocycles with exo-C=N-bonds.

Substrate	Dipole	Reagents Ratio ^a^	Yields, %	Conditions	Note	Refs.
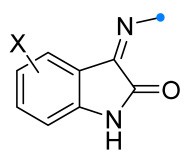	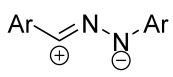	1/2/2	60–95	DCM ^b^, rt, 24 h, N_2_	Dipolarophiles with substituents in indoline aryl fragment (X) were investigated.	[6]
		1/1/24	75–95	CH_3_CN, rt, 18–21 h	X = H. None of the reagents were added gradually.	[44]
		1/1/1	70–90	CH_3_CN, rt, 19–30 h	X = H. Triethylamine was added gradually.	[45] [46]
		1/1/1	78–95	DMF ^b^ or DMA ^b^, MW, 3–5 min	X = H. None of the reagents were added gradually. Reaction mixture was irradiated at 360 W in DMF [45] or at 200 W in DMA [46].	
	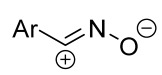	1/2.2/2.6	62–93	Et_2_O/THF (1/1), 0 °C to rt, 3 h	Dipolarophiles with substituents in indoline aryl fragment (X) were investigated. Et_3_N solution was added gradually.	[47]
		1/2/2	54–87	DCM, rt, 5–12 h, N_2_		[49]
		1/2/1	64–86	DCM/acetone, rt, 24 h	Dipolarophiles with substituents in indoline aryl fragment (X) were investigated. None of the reagents were added gradually.	[50]
		1/1/1	77–97	CHCl_3_, 0 °C to rt, 4.5 h	X = H. Dipolarophile with 3-methyl-5-styryl-4-isoxazolylimino-group was investigated. Only C=N-cycloaddition product was obtained (not C=C of styryl). Et_3_N was added gradually upon cooling.	[48]
		1/1/15 ^c^	75–92	DCM, 20 to 40 °C, 1–3 h	X = H. One-pot strategy: dipole precursor was synthesized oxime, NCS ^b^ and pyridine in DCM. Then dipolarophile (in one portion) and Et_3_N solution (gradually) were added.	[51]
		1/1/7	67–87			[52]
		1/1/1	89–96	DMSO, 80 °C, MW, 3 min	X = H. Reaction mixture was irradiated at 400 W.	[52]
		1/1/-	78–95	EtOH, rt, 3 + 3 h	Dipolarophiles with substituents (X) were investigated. One-pot strategy (domino): dipolarophile was generated via condensation, then dipole precursor and DMAP (10 mol %) were added.	[53]
		1/1.1/2	80–85	CHCl_3_, rt, ultrasonication, 20–30 min	Dipolarophiles with substituents in indoline aryl fragment (X). Et_3_N solution was added dropwise.	[54]
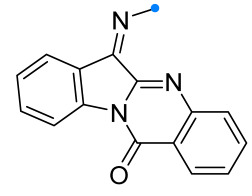	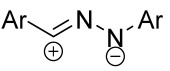	2.2/2.2/2.5	70–93	CH_3_CN, rt, 30 h	Et_3_N solution was added dropwise.	[55]
	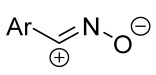	1/1/15 ^c^	76–89	DCM, 20 to 40 °C, 15 h	One-pot strategy: dipole precursor was synthesized oxime, NCS ^b^ and pyridine in DCM. Then dipolarophile (in one portion) and Et_3_N solution (gradually) were added.	[56]
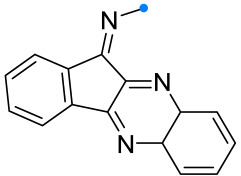	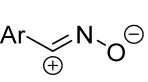	1/1.1/2	80–86	DCM, rt, 15–25 min ultrasonication	Et_3_N solution was added dropwise.	[5]

^a^ The ratio of dipolarophile/dipole precursor/triethylamine, in equivalents; ^b^ See Abbreviations section; ^c^ Reagents ratio 1/3/15 for diimine as dipolarophile.

**Table 4:** Summary of substrates, conditions and yields of the reactions of nitrile imines or nitrile oxides and at exo-C=N bonds of cycles with two heteroatoms.

Substrate	Dipole	Reagents Ratio ^a^	Yields, %	Conditions	Note	Refs.
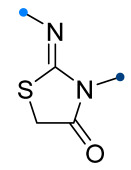	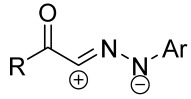	1/1/5	60–87	THF, 0 °C to rt, overnight	Et_3_N solution was added gradually. Only C=N cycloaddition products were formed, C=O bond was inert.	[57]
	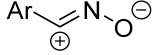	1/1/1	65–85	CHCl_3_, 0 °C, 4–5 h	The same as above.	[58,59,60,61,62,63]
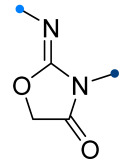	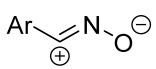	1/1/1	70–82	CHCl_3_, 0 °C, 4–5 h	The same as above.	[60,63]
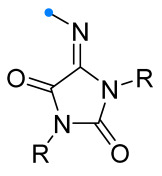	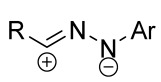	1/1.1/1.2	50–93	DCM, rt, 24 h, Ar	R = methyl, aryl. Et_3_N was added dropwise. Low yield of spiro compound (13%) obtained from p-nitrophenyl substituted dipolarophile.	[64]
		1/1.1	52–90	DCM, rt, 24 h	R = methyl, aryl. Diffusion mixing. Low yield of spiro compound (40%) obtained from p-nitrophenyl substituted dipolarophile.	
	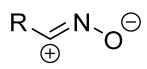	1/1.1/1.2	46–93	DCM, 0 °C to rt, 24 h, Ar	R = aryl. No product when R = COOEt. Et_3_N solution was added dropwise.	[65]
		1/1.1	34–88	DCM, rt, 24 h	R = aryl. No product when R = COOEt. Diffusion mixing.	
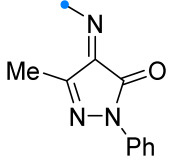	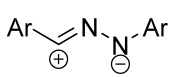	1/1.5	70–97	CH_3_CN, rt, 20 h, light	Light-induced reaction (ultraviolet high-pressure Hg lamp, 365 nm): nitrile imine was generated from tetrazole. Gram scale experiments were conducted.	[66]

^a^ The ratio of dipolarophile/dipole precursor/triethylamine, in equivalents.

**Table 5:** Summary of substrates, conditions and yields of the reactions of nitrile imines or nitrile oxides and at exo-C=N bonds of cycles with three heteroatoms.

Substrate	Dipole	Reagents Ratio ^a^	Yields, %	Conditions	Note	Refs.
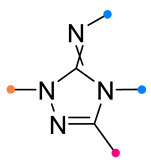	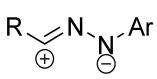	5 ^b^/1/2.9	19–69	C_6_H_6_, rt, 3 d, N_2_	R = Ph, COOEt. Hydrazonoyl chloride was used as a dipole precursor, Et3N was used as a base. Large scale reactions.	[67]
		1 ^b^/2.5/2.5	57–88	DCM, rt, 38 h	R = CHF2, CF3. Hydrazonoyl bromide and K_2_CO_3_ were utilized. No reaction for arylated carbodiimide. Gram scale experiments were conducted.	[68]
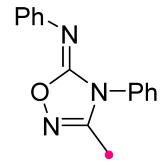	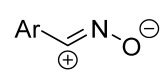	1 ^b^/2 or 1 ^c^/1	50–75	C_6_H_6_, rt to 60–70 °C, 20–30 min	BF3⋅Et2O catalyzed reactions (equal amount relative to dipole). Benzonitrile oxides were used themselves. The method is suitable for carbodiimides as well as independently synthesized substrates.	[71]
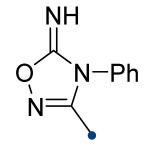	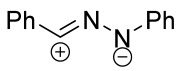	1/1.25/1.3	61–75	THF, rt, 24 h	Hydrazonoyl and hydroxyimidoyl chlorides were used as the dipole precursors, Et3N was used as a base (gradually added). Gram scale experiments were conducted.	[72]
	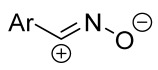	1/1.25/1.3	68–83	THF, rt, 2 h		

^a^ The ratio of dipolarophile/dipole precursor/triethylamine, in equivalents; ^b^ Amount of carbodiimide (cyclic dipolarophile precursor). ^c^ For preliminarily obtained imino-oxazolidine.

## Abbreviations

The following abbreviations are used in this manuscript:

32CA	(3+2)-cycloaddition
DPNI	diphenyl nitrile imine
THF	tetrahydrofuran
DCM	dichloromethane
DMF	N,N-dimethylformamide
DMA	N,N-dimethylacetamide
NCS	*N*-chlorosuccinimide
